# Unexpected response to systemic chemotherapy in case of primarily nonresectable advanced disseminated intrahepatic cholangiocarcinoma

**DOI:** 10.1186/1477-7819-5-36

**Published:** 2007-03-21

**Authors:** Maciej W Slupski, Cezary Szczylik, Milosz K Jasinski

**Affiliations:** 1 Department of Transplantology and General Surgery, Nicolaus Copernicus University, Curie-Sklodowskiej 9, Bydgoszcz, Poland; 2 Department of Oncology CSK WAM, Szaserow 128, Warsaw, Poland

## Abstract

**Background:**

Cholangiocellular cancers account for about 10-15% of primary liver cancers. Prognosis is poor, with expected survival of less than 5% at five-year.

**Case presentation:**

The case described shows remission of a disseminated cholangiocellular carcinoma (focal changes in liver, metastases to lungs) after neoadjuvant chemotherapy. The initial diagnosis was based on ultrasound examination and confirmed with computer tomography. Tumour biopsy and histopathological examination revealed cholangiocellular carcinoma. The patient underwent chemotherapy. After remission of lesions in lungs and reduction/regression of tumours in liver to one focal change, right lobe liver resection was performed. The histopathological examination did not reveal any viable carcinoma cells, only necrotic tissues in place of the primary tumour as well as in local portal vein branches was seen. Thirty months after the operation the patient is in a good overall condition and no recurrence has been observed.

**Conclusion:**

Appropriate neoadjuvant chemotherapy may allow radical resection in a previously unresectable cholangiocellular cancer.

## Background

Intrahepatic cholangiocarcinoma (CCC) is a rare liver malignancy constituting about 10–15% of all primary liver cancers. It is far less common than hepatocellular carcinoma (HCC) which constitute over 80% of primary liver cancers [1,2]. While surgical therapy is the most effective treatment, only 25% of patients are resectable at presentation as CCC is often diagnosed in advanced, nonresectable stages [3,4]. Advanced CCC is associated with particularly poor prognosis, as chemotherapy and radiotherapy have a very limited impact on the disease. We describe a case of CCC that could be resected after downstaging with neoadjuvant chemotherapy.

## Case presentation

In September 2003, a 33-year-old patient was admitted to internal diseases clinic. He had a 2-month history of pain in right upper abdomen radiating to thoracic spine and weight loss (30 kg within 2 months). There were no abnormal signs in physical examination, biochemical tests were within normal range.

**Figure 1:**
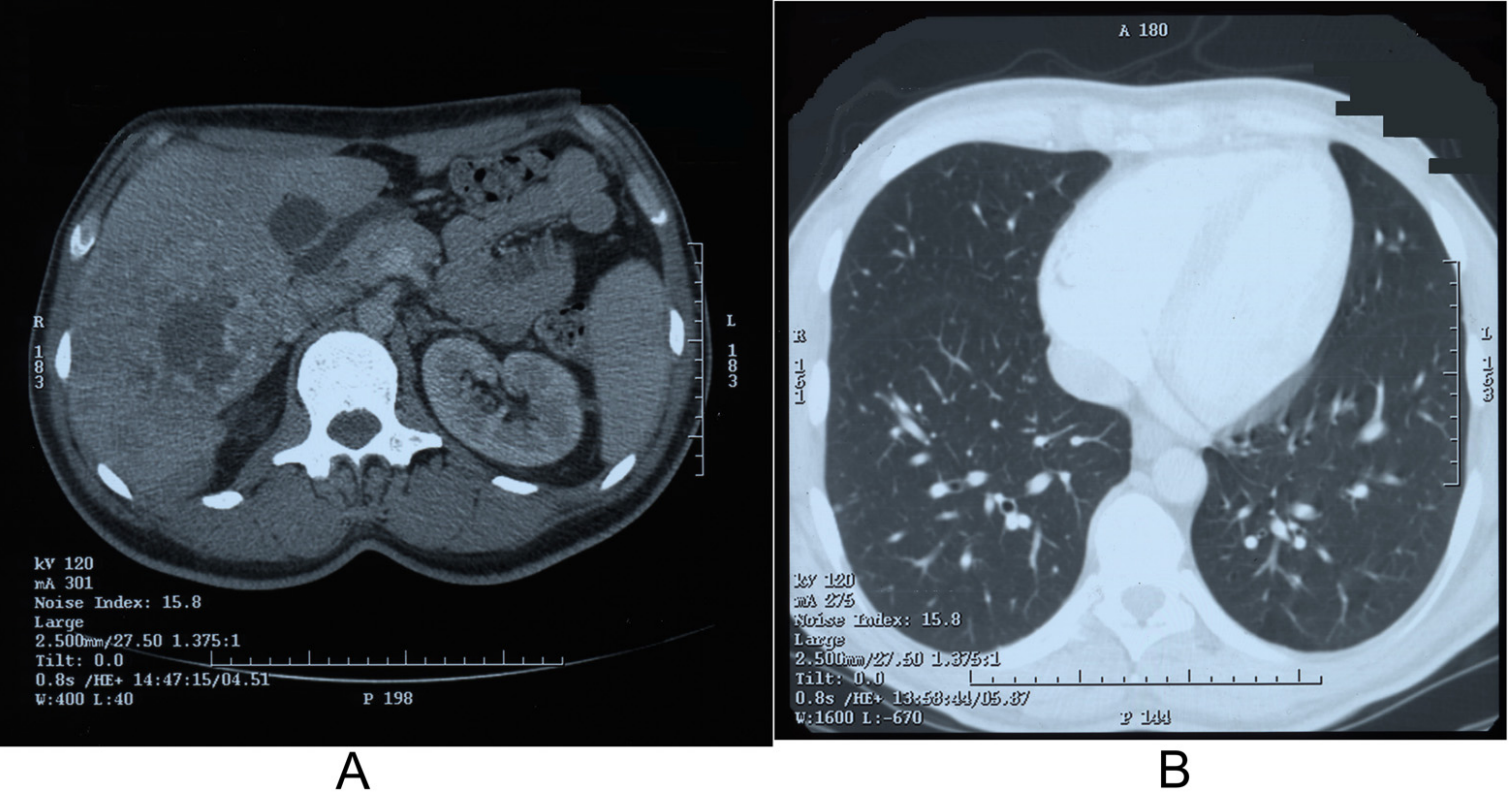
Computed tomography, A – multiple focal changes in liver are visible, B – multiple metastases to lungs are visible.

Computed tomography (CT) revealed multiple focal changes in liver, the largest lesion in the right lobe was 9.2 × 4.5 × 7 cm, and metastases in lungs (Figure 1). Gastroscopy and colonoscopy did not show any digestive tract tumours. Needle biopsy of the tumour showed low-differentiated intrahepatic cholangiocarcinoma. Based on these results, patient was qualified for palliative systemic chemotherapy treatment (PIAF scheme) was started on 29.10.2003. The details of the doses and schedule are given in Table 1.

**Table 1:** Drug name and mode of administration

**date **(day of chth)		
(I day)	1.	Doxorubicin 80 mg i.v.
	2.	Cis-Platinium 40 mg i.v.
	3.	5-FU 800 mg i.v.
	4.	Roferon 9 MU

(II day)	1.	Cis-Platinium 40 mg i.v.
	2.	5-FU 800 mg i.v.
	3.	Roferon 9 M U

(III day)	1.	Cis-Platinium 40 mg i.v.
	2.	5-FU 800 mg i.v.
	3.	Roferon 9 M U

(IV day)	1.	Cis-Platinium 40 mg i.v.
	2.	5-FU 800 mg i.v.
	3.	Roferon 9 M U

PIAF chemotherapy scheme. Patient body surface area 2 m^2^.

5-FU – 5-Fluorouracil

Roferon – Interferon K

In December 2003, after 3 courses of chemotherapy, repeat CT scan showed partial remission of the neoplastic disease, after 6 courses further remission was visible on CT examination.

**Figure 2:**
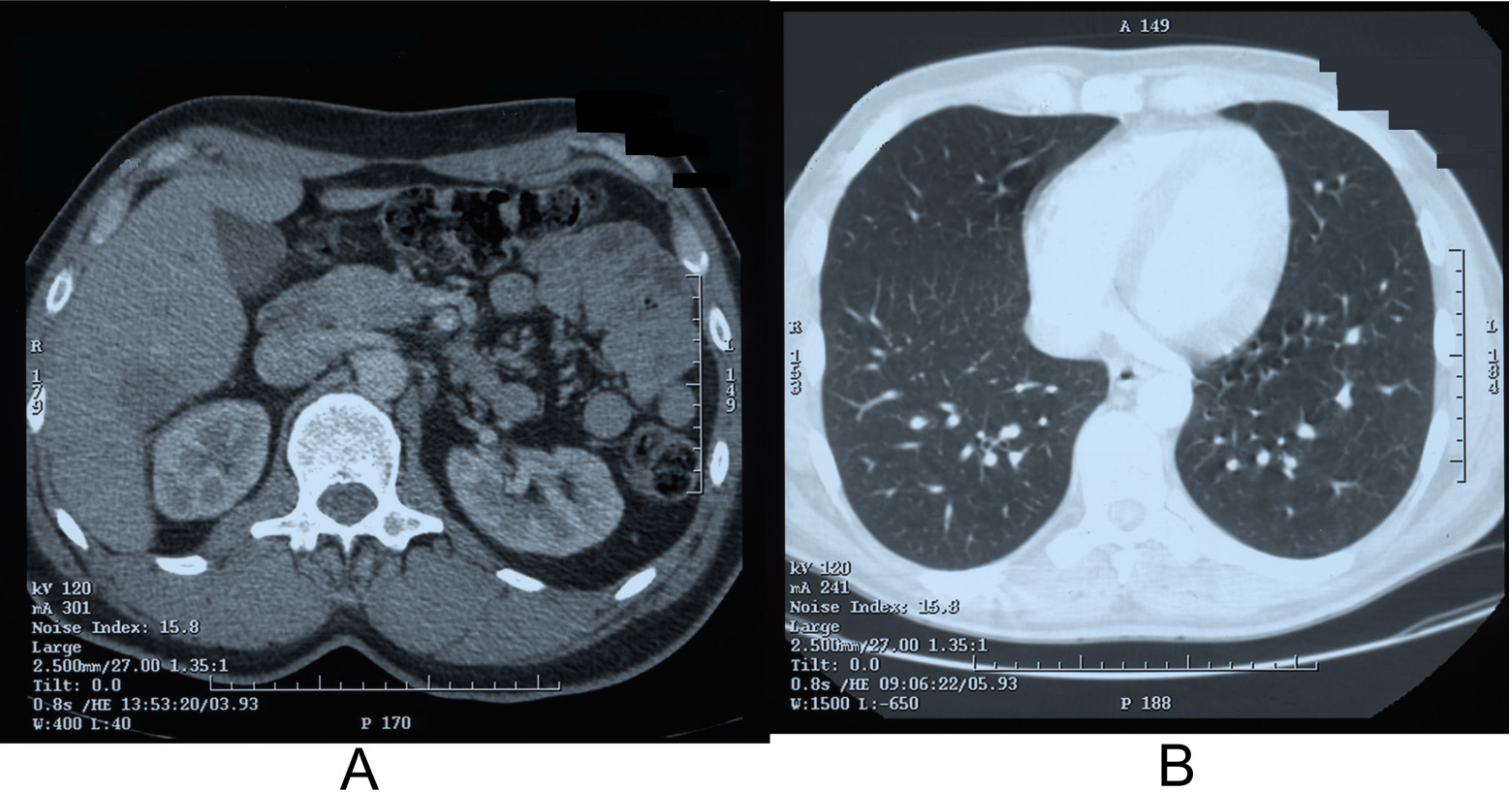
Computed tomography after chemotherapy A – regression of focal changes in liver is visible B – regression of metastases in lungs is visible.

Computed tomography after 9 courses of chemotherapy in May 2004 showed reduction of primary tumour as well as regression of metastases in lungs and other lesions in liver (Figure 2). Patient was qualified for operative treatment and in June 2004, right hemihepatectomy was performed. The postoperative period was without complications, patient was discharged from hospital 8 days after operation in good general condition.

**Figure 3:**
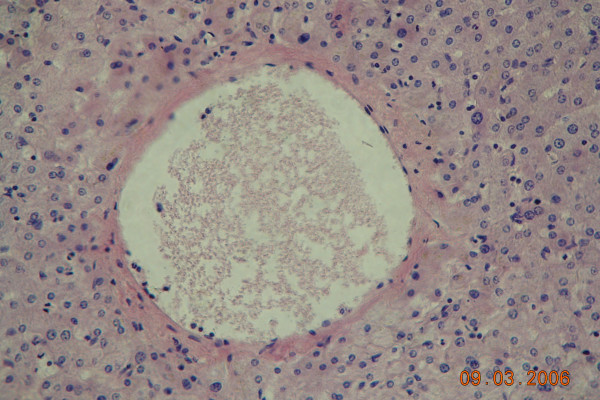
Histopathological specimen of resected liver tumour, 200×. Necrotic masses embolism inside a portal vein branch.

**Figure 4:**
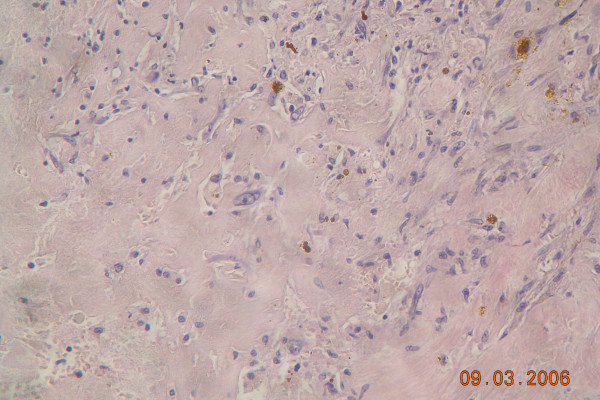
Histopathological specimen of resected liver tumour, 200×. Necrotic masses within the tumour.

Postoperative histopathological examination showed tumour, 26 mm in greatest diameter, encapsulated, with necrotic masses inside capsule. In the surroundings of the tumour necrotic focal change with similar morphology, without capsule was found. No viable tumour cells were found, in surrounding vessels necrotic masses embolisms were found. Morphology suggested cholangiocarcinoma (Figure 3, 4)

After the operation patient received remaining two courses of chemotherapy. Adverse effects included mild leucopenia, thrombocytopenia (80000/μl), mild increase in alkaline phosphatase (200 U/l) and alopecia. Repeat CT scan in August 2004 showed regeneration of liver parenchyma, there was no sign of recurrence of the neoplasm. Control examinations 12, 18, 24 and 29 months after operation did not show any signs of recurrence.

## Discussion

Resection is the preferred treatment in the management of CCC; patients with resected CCC are the only long-term survivors [5-8]. The precise results differ depending on tumour stage, general condition of the patient and non-operative method applied. Chemoembolization gives better results than systemic chemotherapy, however, in patients with disease as advanced as the described case it is not applicable. Patients with metastases are considered to have particularly poor prognosis, median survivals in such cases is below 8 months. [9]

Effectiveness of chemotherapy is unsatisfactory - less than 30% responses to treatment, usually it does not significantly improve the prognosis. Currently, phase III clinical trials using Gemcitabine had been conducted and collected data allows expecting more encouraging results. [9-11]

Various chemotherapy regimes based on 5-FU in combination with cisplatin, interferon, and doxorubicin were reported to be active in CCC [12-15]. Although chemotherapy regimens containing platinum analogs have reported higher response rates (20–40%), their toxicity is considerable, especially myelosuppression and gastrointestinal upset [16,17]. PIAF regimen is reported to produce some dramatic anti tumour responses, yet it is uncertain if it can be indicated for all patients with cholangiocarcinoma because of its toxicity [18]. The case described presents advanced cholangiocarcinoma. Numerous focal changes in liver and metastases to lungs visible in computed tomography indicates that the process was highly advanced. TNM staging was T4 Nx M1 (IVb). Performing a curative resection was impossible in such an advanced stage. The chemotherapy regimen was chosen as a salvage treatment considering young age of the patient and advanced stage of the neoplasm. Systemic chemotherapy induced total remission of metastases in lungs and reduction of changes in liver to one tumour in right lobe, which allowed radical resection. It is uncertain why such a good response to chemotherapy occurred in case of tumour considered to be chemoresistant. PIAF regimen was relatively well tolerated by the patient, which can be attributed to his young age and good overall condition.

Necrosis was the only remaining focal change in liver along with presence of necrotic masses in portal vein branches around the tumour – this can be considered as a proof of chemotherapy effectiveness. Thirty months after operation patient is in good general condition and shows no sign of disease recurrence.

## Conclusion

The unusually good response of the neoplasm to chemotherapy is worth reporting, especially because systemic chemotherapy is considered little or non effective in disseminated cholangiocellular carcinoma and there is still no established protocol for it. Although this chemotherapy regimen can not be recommended as a treatment in case of CCC basing on a single case report, this patient's response shows that therapy of an advanced CCC is possible. This case may suggest that the chemotherapy scheme described could be considered as a therapy option for certain patients with advanced cholangiocellular carcinoma, possibly young ones who are more likely to tolerate the toxicity of this regimen.

## Competing interests

The author(s) declare that they have no competing interests.

## Authors' contributions

**MS **– carried out surgical procedure, provided data about surgical procedure and follow-up of the patient, helped to draft the manuscript

**CS **– carried out chemotherapy and provided data about chemotherapy treatment

**MJ **– drafted the manuscript, collected histopathological and radiological data

All authors have read and approved the manuscript.
